# A systematic review of rodent pest research in Afro-Malagasy small-holder farming systems: Are we asking the right questions?

**DOI:** 10.1371/journal.pone.0174554

**Published:** 2017-03-30

**Authors:** Lourens H. Swanepoel, Corrie M. Swanepoel, Peter R. Brown, Seth J. Eiseb, Steven M. Goodman, Mark Keith, Frikkie Kirsten, Herwig Leirs, Themb’alilahlwa A. M. Mahlaba, Rhodes H. Makundi, Phanuel Malebane, Emil F. von Maltitz, Apia W. Massawe, Ara Monadjem, Loth S. Mulungu, Grant R. Singleton, Peter J. Taylor, Voahangy Soarimalala, Steven R. Belmain

**Affiliations:** 1 Department of Zoology, University of Venda, Thohoyandou, South Africa; 2 ARC-Institute for Soil, Climate and Water, Pretoria, South Africa; 3 Commonwealth Scientific and Industrial Research Organisation, Canberra, Australia; 4 Department of Biology, University of Namibia, Windhoek, Namibia; 5 Association Vahatra, BP, Antananarivo, Madagascar; 6 Field Museum of Natural History, 1400 South Lake Shore Drive, Chicago, IL, United Statets of America; 7 Centre for Wildlife Management, Department of Animal and Wildlife Sciences, University of Pretoria, Private Bag X20 Hatfield, Pretoria, South Africa; 8 ARC-Plant Protection Research Institute, Pretoria, South Africa; 9 University of Antwerp, Groenenborgerlaan, Antwerp, Belgium; 10 Department of Biological Sciences, University of Swaziland, Private Bag 4, Kwaluseni, Swaziland; 11 Pest Management Centre, Sokoine University of Agriculture, Morogoro, Tanzania; 12 Crop & Environmental Sciences Division, International Rice Research Institute, Metro Manila, Philippines; 13 South African Research Chair on Biodiversity Value & Change, University of Venda, Thohoyandou, South Africa; 14 Natural Resources Institute, University of Greenwich, Chatham Maritime, Kent ME4 4TB, United Kingdom; University of Sydney, AUSTRALIA

## Abstract

Rodent pests are especially problematic in terms of agriculture and public health since they can inflict considerable economic damage associated with their abundance, diversity, generalist feeding habits and high reproductive rates. To quantify rodent pest impacts and identify trends in rodent pest research impacting on small-holder agriculture in the Afro-Malagasy region we did a systematic review of research outputs from 1910 to 2015, by developing an *a priori* defined set of criteria to allow for replication of the review process. We followed the Preferred Reporting Items for Systematic Reviews and Meta-Analyses guidelines. We reviewed 162 publications, and while rodent pest research was spatially distributed across Africa (32 countries, including Madagascar), there was a disparity in number of studies per country with research biased towards four countries (Tanzania [25%], Nigeria [9%], Ethiopia [9%], Kenya [8%]) accounting for 51% of all rodent pest research in the Afro-Malagasy region. There was a disparity in the research themes addressed by Tanzanian publications compared to publications from the rest of the Afro-Malagasy region where research in Tanzania had a much more applied focus (50%) compared to a more basic research approach (92%) in the rest of the Afro-Malagasy region. We found that pest rodents have a significant negative effect on the Afro-Malagasy small-holder farming communities. Crop losses varied between cropping stages, storage and crops and the highest losses occurred during early cropping stages (46% median loss during seedling stage) and the mature stage (15% median loss). There was a scarcity of studies investigating the effectiveness of various management actions on rodent pest damage and population abundance. Our analysis highlights that there are inadequate empirical studies focused on developing sustainable control methods for rodent pests and rodent pests in the Africa-Malagasy context is generally ignored as a research topic.

## Introduction

World hunger and food insecurity are principally linked to poverty [1, 2]. The majority of the rural poor are dependent on farming where 90% of farm sizes are less than two hectares [3]. Furthermore, up to 80% of undernourished people live in countries where the majority of farming occurs under such small-holder farming practices [4]. As such, small-holder farmers are the backbone of global food security [5]. While rural small-holder farmers face several social and environmental problems in food production [6], agricultural pests are a major factor in yield gaps pre-harvest and losses post-harvest [7, 8]. Rodent pests are problematic in terms of agriculture and public health since they can inflict considerable economic damage [9, 10], because of their abundance, diversity, generalist feeding habits and their high reproductive output [11].

In Asia, under traditional rice farming systems, rodents typically cause chronic losses to rice in the order of 5–10% per annum [12], while episodic population outbreaks cause severe losses that place at risk the food security of entire communities [13]. In Tanzania, chronic pre-harvest losses to maize are around 15%, while damage at sowing and to seedlings can exceed 40% [14]. Population irruptions of *Mastomys natalensis* have caused yield losses up to 48%, and during acute outbreaks, damage has reached 80–100% of sowing and seedling stages in maize [14, 15]. In Kenya, rodents have caused losses of 20–30% to maize crops, with 34–100% during rodent outbreaks [16]. In parts of South America, native rodents cause crop damage varying between 5 and 90% of total production [17]. Post-harvest losses caused by rodents in grain stores add around 5–14% each year to the losses of small-holder families [18, 19].

Meerburg et al. [20] conservatively estimated that if rodent losses to rice production could be reduced by 5% this would save 70 million tonnes of rice, which is sufficient to provide the annual food consumption for almost 280 million people in developing countries, i.e. enough to feed 34% of the total undernourished people in the world. Thus, controlling rodent numbers to prevent subsequent losses remains one of the key strategies to secure long-term food security, agro-ecological sustainability and economic development, especially among small-holder farmers. Rodents are also an important public health issue [20, 21], but this aspect is not covered in this review.

Several methods are employed globally to control rodent pests [22], even though definitive and sustainable solutions seem currently unattainable through poor application of improved management strategies and limited technology development [23]. The paradigm of Ecologically-Based Rodent Management has gained momentum over the past 20 years as an alternative, effective and sustainable rodent control concept [23, 24]. Several research and community-based development programmes have addressed several aspects of EBRM and its implementation in rural communities [24–27]. These projects have been successful in raising awareness [26] about the seriousness of rodent pests, implementing several rodent control campaigns, as well as highlighting the challenges related to sustainable and effective control [9]. While some reviews have emphasised the importance of rodent pests and their control in the developed world [22], and others have summarised the impact and control of pest rodents in southeast Asia small-holder agricultural areas [9, 12, 28], no study thus far has reviewed the impacts of rodents on small-holder farming in Africa. This is unfortunate for two reasons, first improving food security in the African-Malagasy small-holder industry can have large outcome effects on community well-being. Secondly, several rodent pest control actions are often suggested, with little research of the effectiveness of such approaches. For example, evaluating the effectiveness of intervention actions often requires complicated replicated experimental designs to test hypotheses about rodent population dynamics and associated crop damage [23, 25, 29]. It is, therefore, important to assess the current and historical approaches to pest rodent research in Africa to enable researchers to evaluate the effectiveness of current research approaches and to develop more appropriate research protocols if needed. Furthermore, such a review will highlight current and historic trends of rodent pest research, and its impact on small-holder agriculture and will enable researchers and governments to evaluate current and future control strategies. The aims of this study are firstly to establish the current state of knowledge for rodents in the agricultural sector, with an emphasis on Africa and Madagascar, by systematically reviewing research on rodents including their occurrence, damage and control and, secondly, to highlight important research gaps at continental and national scales.

## Material and methods

### Selection of studies

Our initial intention was to collate all relevant studies in a meta-analysis on rodent damage in the agricultural sector and effectiveness of practiced control measures. However, very few publications have focused on this subject. Hence, instead of a full meta-analysis, we compiled a review of published publications (and reports) in this review, to present the *status quo* on rodent research in agricultural systems in the Afro-Malagasy context, highlighting gaps and future research needs. We focused on the spatial and temporal distribution of rodent studies, extent of rodent damage, and the methods and effectiveness of their control.

**Fig 1:**
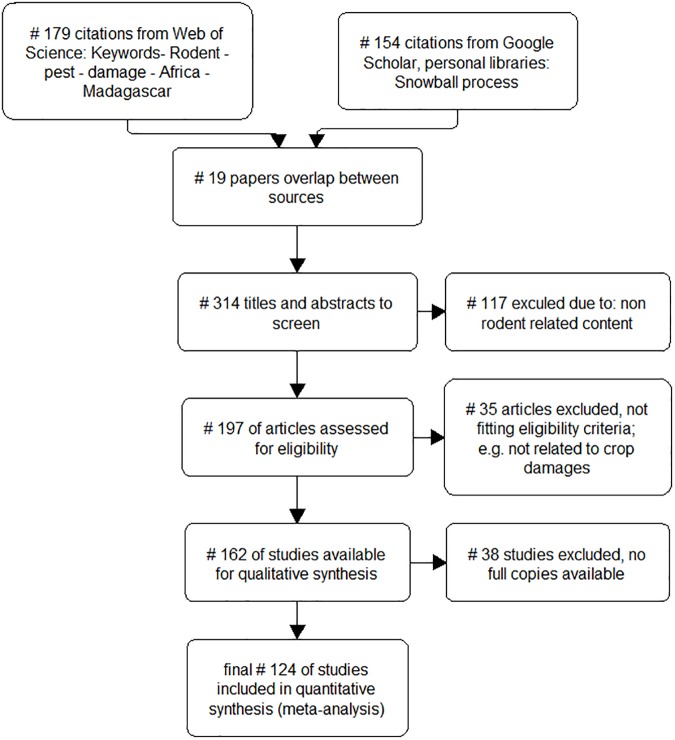
PRISMA statement for the publications retained in the review.

To quantify rodent pest impact and identify trends in rodent pest research on small-holder agriculture in the Afro-Malagasy context we conducted a systematic literature review covering the period 1910–2015. We developed an *a priori* defined set of criteria to allow for replication of the review process [30] and followed the Preferred Reporting Items for Systematic Reviews and Meta-Analyses (PRISMA statement and Checklist) guidelines in recording publications excluded or included during screening stages (Fig 1 and S1 Table; [31, 32]. We first searched the Web of Science ^TM^ database and focussed on peer-refereed journal publications and book chapters since these are systemically accessible and normally indicative of scientific progress in a particular field. We then used a snowball process where we scanned the references of appropriate publications to discover additional publications, reports and grey literature [33]. Finally, we supplemented the publications from the various co-authors’ personal libraries.

The review was geographically limited to continental Africa and Madagascar and we only selected publications that described the effect of rodent pests on small-holder agriculture. This was achieved by searching online databases for publications that contained the following keywords in the topic: ‘Rodent pest damage’, ‘Africa’ and/or ‘Madagascar’. Abstracts of relevant studies were screened using the ‘metagear’ R package [32] to compile a list of appropriate publications matching the review criteria. We finally only retained publications for which we could source the full text version for quantitative data analysis (S1 List). Search history data retrieved from Web of Science can be found in S2 List and S1 Web of ScienceTM saved search (AU file).

Each paper was categorised using the following criteria: i) review publications that summarised knowledge regarding rodent pests in the Afro-Malagasy context; ii) field studies reporting rodent damage and/or ecology in agriculture; iii) knowledge, attitudes and practice (KAP) that interviewed local residents regarding rodent pests and associated damage (including traditional questionnaire studies–we classed all questionnaires and KAP as ‘interviews’). We designated more than one research category to a particular study (e.g. interview [KAP] studies were often paired with field trials; [34] when overlapping occurred.

We extracted the following information from each publication where available: spatial data (country and location of study sites), temporal data (publication date), size of study area, rodent ecological data (rodent species, densities, species composition), and agricultural data (crops and crop stages). To assess rodent impact and management approaches we extracted several crop impact matrixes. Extracting crop impact data was however complicated by several factors. First, the correlation between rodent crop damage and subsequent yield losses are not necessarily linear [35]. This is important since some crops compensate for early crop damage (e.g. during seedling stage) and subsequent yield losses would not necessarily correlate to these early damage estimates [35]. Secondly, the estimation and quantification of rodent crop impact varied considerably between different studies, crops, cropping stages and field methods. In order to standardize and improve the credibility of extracted crop loss data we restricted our analysis to crop loss data collected during field studies. We excluded crop loss data reported in reviews (since these are essentially a repeat of field data) and interview studies. We also restricted crop loss data to mature crop stages and storage and highlight that impact during the seedling stage should be viewed as damage and not necessarily crop losses. However, since levels of seedling damage almost equal subsequent crop losses, seedling damage is a good proxy for crop loss (e.g. for temperate crops, 10% seedling losses resulted in 9% crop loss at harvest; [36].

We considered all crops and standardized data extraction to only two growth stages (seedling, mature [defined as all stages]) plus storage losses. We used both ranges in crop losses (e.g. 10%-20% losses) and point estimates in presenting rodent crop impact. We further grouped crop losses into 0–20%, 20–50% and >50% bins. We followed this approach since a large proportion of crop losses were reported as ranges (e.g. up to 20% loss), rather as point estimates. We grouped publications into two broad research themes, those that emphasised applied or basic research. Applied research included publications that actively investigated methods to control rodent pest damage and populations, or at least related rodent abundance to damage levels, which included: 1) before and after intervention studies [damage and/or rodent densities]; 2) damage management publications [e.g. relating rodent abundance to crop damage, or comparing control methods]; and 3) mathematical modelling of crop damage to inform management. Basic research included publications that only reported results, and did not relate rodent abundance or crop damage levels to management interventions, which included: 1) rodent ecology [e.g. population ecology, movement ecology, genetics]; 2) crop damage/yield loss estimates; 3) interview studies; and 4) and reviews.

### Data analysis

Effect sizes like Hedges’d or ln(R) are often used to quantify the direction and magnitude of experimental impact [37]. However, none of the studies in this review qualified as replicated studies and we, therefore, could not estimate within-study variance. Furthermore, only seven studies (4%) reported before and after intervention estimates. We, therefore, used *ln*(X_e_/X_c_), where X_e_ and X_c_ are the mean estimates for intervention and control, respectively, to estimate manipulation effect. *ln*(X_e_/X_c_) > 0 means that intervention resulted in a higher response (e.g. higher density of rodents post intervention), *ln*(X_e_/X_c_) < 0 indicated a negative response (e.g. lower rodent density) while 0 indicates no response [29]. We extracted values for X_e_ and X_c_ from graphs, tables or text.

We used the Chi-Square test to investigate effect of country on research theme, research theme per country and number of studies per country [38], the Mann-Kendall to test for a significant temporal trend in research publications [39], the t-test to test for significant mean differences between paired treatments and Kruskal-Wallis for difference in crop losses [40]. All data analysis was done in R [41], and we used the ‘metagear’ package [32] to draw the PRISMA flow diagram and ‘Kendall’ package for analysis of research trend [42]. We used QGIS 2.18.3 [43] to produce the distribution map and took political boundaries (Africa and Madagascar) from www.Naturalearthdata [44].

## Results

### Temporal and spatial

**Fig 2:**
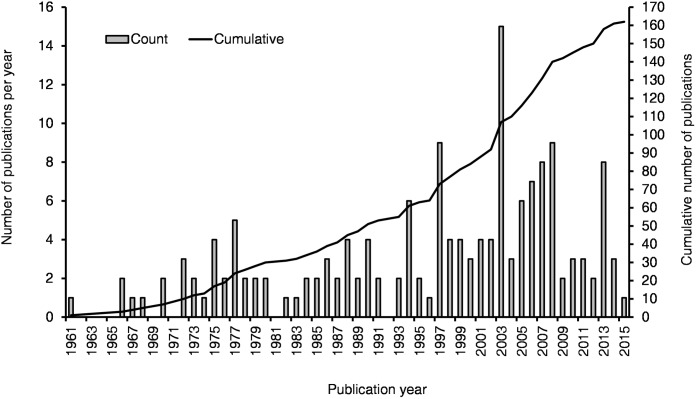
Temporal trend of rodent pest studies in Afro-Malagasy agricultural systems from 1960 to 2015.

A total of 162 publications (Fig 1) met the criteria of being suitably focussed on rodent pest damage in Afro-Malagasy agricultural systems. During 2003 there were an unusually high number of publications recorded (n = 15), resulting from the publication of a conference proceedings dedicated to this topic (“Second International Conference on Rodent Biology and Management”, [28]. No publications were found prior to 1960, and we subsequently on use the period post 1960 in analysis and graphs. Using publication date as an indication of a temporal trend, a mean of 3.44 (± 1.66) publications were published per annum, with a significant but small increase in publication rate over the time period (Mann-Kendall test: tau = 0.369, *p* < 0.0001; Fig 2).

**Fig 3:**
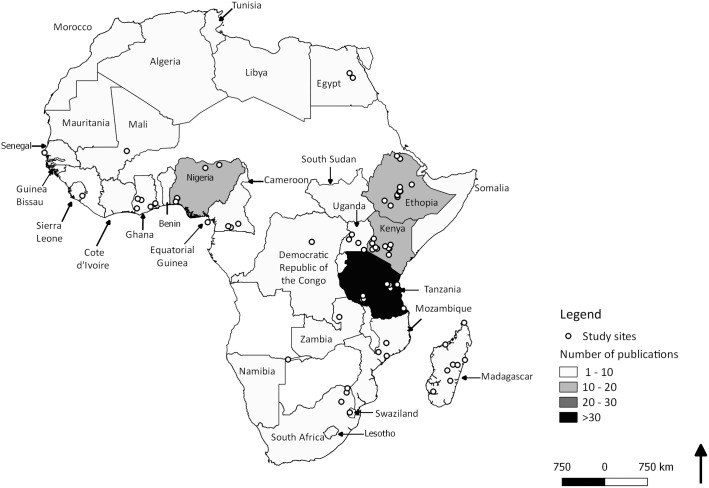
Spatial distribution of rodent studies in the Afro-Malagasy agricultural systems during the period 1960–2015. Map is limited to countries in which rodent pest studies were registered and shading represents number of studies (see legend). Map created by LHS.

Whilst rodent pest research has been conducted across much of the African continent (32 out of 48, countries (including Madagascar) registered at least one relevant study), there was a disparity in the number of studies per country (χ^2^_18_ = 205.72, *p* < 0.0001; Fig 3). Research was focused in nodes in East and West Africa. Specifically four countries; Tanzania (24.69%), Ethiopia (8.64%), Nigeria (8.64%), and Kenya (8.02%), accounted for 50% of all rodent pest research on the continent (Fig 3). The high research intensity in Tanzania is due to the Pest Management Centre at the Sokoine University of Agriculture, with a strong focus on rodent pests in agricultural systems. Similarly, individual studies per country clustered around established research sites (Fig 3).

### Research themes

To extract quantitative data and assign an appropriate research theme to each paper, we had to have access to the full text of each paper. However, for 38 out of 162 (23%) publications we could not source full texts, due to limited publication as unpublished theses or in local (and often discontinued) journals. These publications were, therefore, excluded from all subsequent analyses but are reported in S1 List, and only the remaining 124 full text publications were used for further analysis.

**Fig 4:**
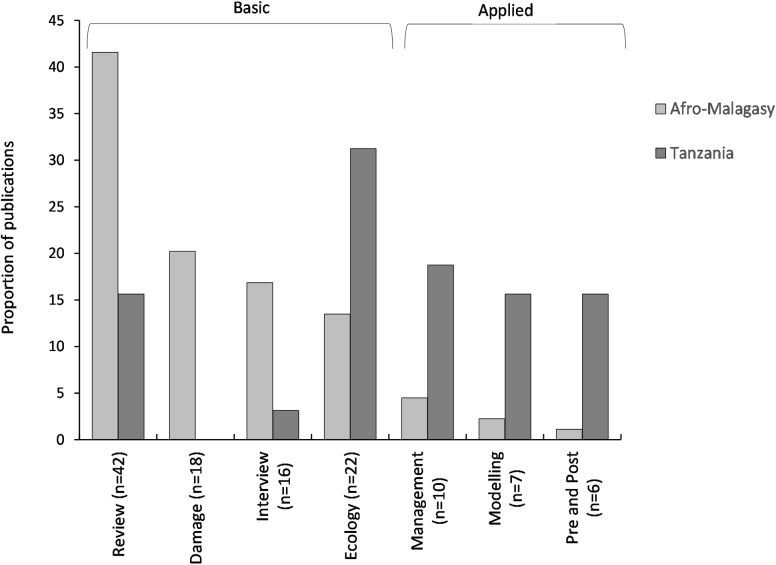
Proportional breakdown by research theme of studies on rodent pests in Tanzania and other Afro-Malagasy countries published between 1960 and 2015.

There was a significant difference in the number of publications in the different categories (χ^2^_4_ = 68.28, *p* < 0.0001), with the majority of studies being field trials (44.72%), followed by reviews (43.96%), interview studies (8.13%), mathematical models (8.13%) and a combination of field trials and interview studies (4.07%). Since the majority of publications originated from Tanzania (26.02%), we benchmarked research from the rest of Afro-Malagasy countries to Tanzania. There was a disparity in the research themes addressed by Tanzanian rodent pest publications compared to publications from the rest of the Afro-Malagasy areas (χ^2^_20_ = 194.19, *p* < 0.001). Research in Tanzania had a more applied focus (50% of publications) compared to the rest of the Afro-Malagasy agricultural systems that had a more basic research approach (92% of publications; Fig 4). Furthermore, Tanzanian basic research was dominated by ecological studies of pest rodents (31% of publications; Fig 4). In contrast, research from the rest of the Afro-Malagasy countries focused on interview studies (17% of publications), while review studies (41% of publications) played an important role in providing rodent pest information. Other Afro-Malagasy studies also tended to focus on damage assessments (20% of publications) and rodent pest ecology (13% of publications; Fig 4). Applied research appears to be more established in Tanzania with damage intervention research (pre- and post-intervention studies (16%), mathematical modelling (16%) and management research (19%) contributing to the high levels of rodent pest research (Fig 4).

### Rodent species involved

A total of 127 rodent species were detected during pest related research in the Afro-Malagasy agricultural systems (S2 Table). However, pest research was dominated by only 10 species (Table 1) of which *Mastomys* spp. (57% of studies), *Arvicanthis* spp. (34%) and *Rattus* spp. (31%; Table 1) were the most important species.

**Table 1:** Rodent species as reported in African-Malagasy rodent pest research (1910–2015).

Species list	Nr of Publications	Proportion of publications
*Mastomys*	70	0.57
* Mastomys natalensis*	*51*	*0*.*41*
* Mastomys erythroleucus*	*10*	*0*.*08*
* Mastomys* spp.	*9*	*0*.*07*
*Arvicanthis*	42	0.34
* Arvicanthis niloticus*	*32*	*0*.*26*
* Arvicanthis* spp. (others)	*10*	*0*.*08*
*Rattus*	38	0.31
* Rattus rattus s*.*l*.	*26*	*0*.*21*
* Rattus* spp.	*12*	*0*.*10*
*Mus*	27	0.22
* Mus musculus*	*8*	*0*.*07*
* Mus minutoides*	*6*	*0*.*05*
* Mus mahomet*	*5*	*0*.*04*
* Mus* spp.	*8*	*0*.*07*
*Lemniscomys*	14	0.11
* Lemniscomys striatus*	*10*	*0*.*08*
* Lemniscomys* spp.	*4*	*0*.*03*
*Gerbilliscus* spp.	13	0.11
*Cricetomys* spp.	11	0.09
*Meriones* spp.	10	0.08
*Thryonomys* spp.	9	0.07
*Xerus* spp.	9	0.07

### Crop impact

Extracting meaningful information on crop losses caused by pest rodents proved to be challenging. Nonetheless, 34% of studies provided some data on crop damage. From these studies, we excluded the review and interview studies since these did not always describe their method of damage estimation, which included several interview studies where damage were quantified by severity, rather than giving damage estimates.

**Fig 5:**
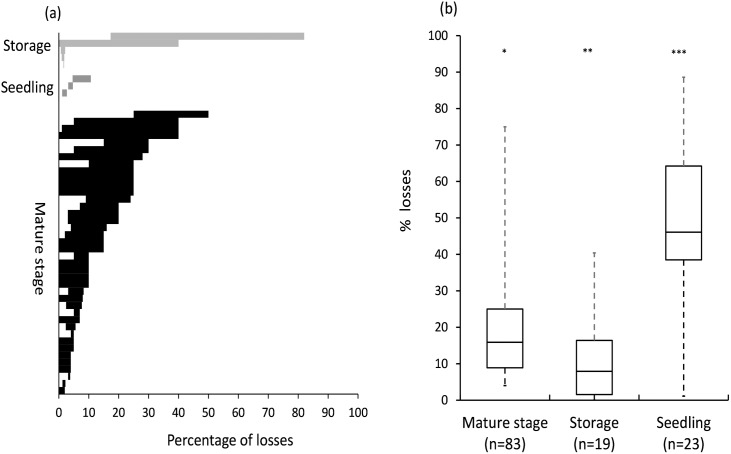
Range of crop losses (a) and median point estimates for crop losses (b) as reported for different crop stages and storage. Asterisks denote significant differences, whiskers minimum and maximum values, box plot indicate third and first quantile and median. n = number of data points.

**Fig 6:**
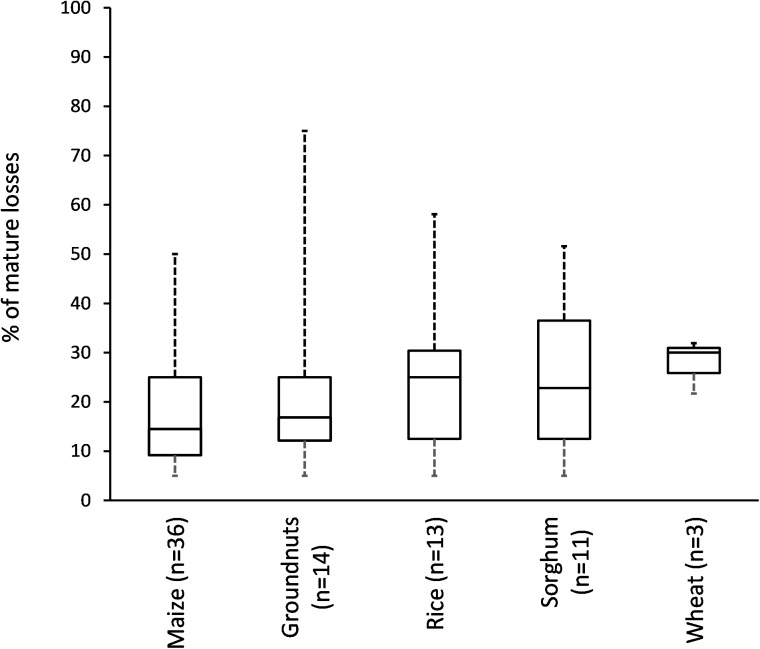
Median crop losses for different crops at maturity stage. Whiskers represent minimum and maximum values, box indicate first quantile, third quantile and median while n = number of data points.

There were large variations in crop loss estimates between the different growth stages and storage, as well as between losses reported as range values or point estimates (Fig 5A & 5B). Median crop losses were significantly different between crop stages and storage (H = 23.25, df = 2, p<0.0001) and the highest losses were recorded during the seedling stage (Fig 5B). The largest proportion of seedling loss studies (100%) reported losses below 50%. Crop losses during maturity varied considerably (0%-50%; Fig 5A); however the majority of losses (68%) fell between 20%-50%. Median mature crop losses (15.9%; Fig 5B) were lower than median storage losses (7.9%; Fig 5B). We found no significant difference in losses for the different crops (H = 4.32, df = 4, *p* = 0.365; Fig 6), probably due to large variation in crop loss estimates. Overall rodent damage was reported for 46 different crop species (S3 Table); however, maize dominated publications (22%) followed by rice (8%) and wheat (7%). In terms of cropping systems, durable commodities dominated research (56% of publications), followed by root vegetables (10%) and fruit trees and vegetables (7% respectively).

### Rodent control and interventions

Various methods were used to control rodent pests, which could be divided into four broad categories: habitat management, chemical control, predation, and trapping. The most important actions to control rodent pests was habitat management (25%), followed by trapping (15%), chemical control (17%), predation (15%; which included dog [2%] and cat predation [%] and avian predation), and other (22%), which include acoustic scaring, glue, guarding, sanitation, praying and charms. Habitat modification included burning of grass, ploughing, inter-cropping and fencing.

We could only source seven studies (8%) that investigated the efficacy of a management intervention on rodent pests and damage. However, all these studies lacked proper replication preventing us to draw appropriate conclusions about the effectiveness of management interventions, even though individual studies suggested that all intervention actions resulted in declining rodent pest damage and abundance (Table 2). For example, ploughing (n = 3 studies) had a significant but small negative effect on rodent abundance, which declined 1.45-fold after ploughing (t-test, mean *ln* (X_e_/X_c_) ± 95% CI = -0.34 ± 0.52, t [2] = 4.19, p = 0.03).

**Table 2:** Treatment effect for individual rodent studies involving certain intervention actions based on reviewed publications

	Losses before^1^ (X_c_)	Losses after^2^ (X_e_)	Effect	Magnitude
	X_c_	X_e_	Effect	
Intervention method	%	%	ln(X_e_/X_c_)	x-fold
Barriers/fencing	12.6	9.6	-0.27	1.31
Trapping^6^	12	4	-1.03	2.79
Proper storage protection	40.4	7.9	-1.63	5.11
	Abundance^3^: Before^4^	Abundance^3^: After^5^		
Chemical control	903	225	-1.39	4.01
Mono crop: ploughing	114.9	83.9	-0.31	1.37
Intercrop: ploughing	151.4	138.8	-0.09	1.09
Ploughing	60	32	-0.63	1.88
Inter vs mono crop	87	69	-0.23	1.26

^1^ Crop losses as measured before an intervention which include no-fencing/barriers, no trapping and inadequate storage

^2^ Crop losses as measured after intervention which included fencing/barriers, trapping and improved storage

^3^ Abundance defined as trap success is used as a proxy for rodent abundance

^4^ Abundance as measured before the intervention which include no ploughing, no chemical control and inter-cropping

^5^ Abundance measured after intervention which include ploughing, chemical control and mono-cropping

^6^ Mean from two trapping intervention studies

## Discussion

### Damage estimates

This analysis has highlighted that pest rodents do indeed have a significant negative effect on Afro-Malagasy small-holder farming communities. Even though damage estimates varied considerably between crop stages, in the methods used to estimate damage, and in geographical scope, there appears to be considerable support that total crop losses due to rodent pests remain around 15%. Interestingly, our observed crop loss estimates closely concur with simulated rodent grazing models for Australian crop systems showing a mean yield loss = 12.4% [35]. These losses are much higher than damage levels farmers are willing to tolerate, usually around 5% [35], which emphasises the large impact rodent pests can have on agricultural production. Rodent damage varied between growth stages, and damage during the seedling stage can be extensive [45]. We found that the majority of studies reported seedling losses in excess of 50% damage, which indicated that the greatest rodent pest impacts often occurs during seed emergence, particularly for maize [46]. High seedling losses have also been observed to lead to high crop losses for both rice [47] and wheat [48]. It has been suggested that the percentage seedling loss is a good predictor of subsequent crop losses (e.g. 10% seedling losses resulted in 9% crop loss at harvest; [36].

Whilst we could extract some meaningful crop loss data, estimated losses from the majority of studies was not insightful. For example, some studies did not distinguish between rodent damage and other agricultural pests [49, 50]. Other studies report on general agricultural losses that occurred during planting, harvesting, and post-harvest processes, of which rodents were only one of many agents [50, 51]. Extracting estimated losses from interview studies was particularly problematic since these studies were often only interested in perception of damage, or severity [52]. Respondents also were not always able to estimate losses [34], suggesting that estimated losses based on interview data might be significantly biased.

Standardized methods for estimating rodent impact were not generally followed by the majority of studies. Whilst the robustness of a standardized approach in estimating rodent damage has been developed for maize [53], not all studies employed these methods. Furthermore, standardized methods in estimating damage during other crop stages and storage seem not to be well established. Unless damage estimates are standardized for crop stages, it remains difficult to make meaningful conclusions and comparisons between estimates, interventions and countries. Furthermore, relating damage caused to crops to subsequent yield loss was not adequately addressed in the majority of the publications. Since not all damages relate to yield losses [35] it is imperative to establish the relationship between crop damage and losses at various cropping stages.

An important relationship exists between crop damage and rodent density [35, 54]. As such, crop damage will vary by rodent abundance; thus monitoring rodent abundance should be an important variable in initiating control actions [35]. Field studies and simulation work has established this relationship as sigmodal where increased damage is observed when rodent densities at the seedling stage increase above 20 animals/ha for *Mastomys natalensis* [55] and around 100 animals/ha for the house mouse *Mus domesticus* [35]. However, some studies have highlighted confusion between rodent damage rates and germination failure rates [55] and most studies rarely take into account germination failure rates in assessing rodent damage at the seedling stage. Given the importance in the relationship between rodent abundance and damage, it is surprising that almost no studies have investigated these relationships to understand when rodent damage exceeds acceptable levels. Such information is of vital importance in setting rodent pest monitoring protocols and determining the initiation of intervention actions.

### Effectiveness of rodent control

There was a paucity of research related to the impact and effectiveness of management actions. Habitat management or modification was the most used method in trying to control rodent abundance and damage. While our analyses showed that ploughing produced meaningful and detectable reductions in rodent abundance, the robustness of such a conclusion is limited by low sample sizes and lack of replication. Similarly, communal trapping has been shown to be an effective method to reduce rodent density to acceptable levels [56, 57]. Such non-chemical control methods are important since several studies have highlighted the limitations of relying on chemical rodent control, especially for resource poor farmers [23]. For example, chemical control may reduce numbers initially, but surviving animals will compensate with higher survival and breeding success [58, 59]. This is important since several mathematical models have highlighted that control efforts are relatively ineffective when interventions occur at high rodent abundances, which is normally when chemical control is applied [60, 61]. The effectiveness of chemical control is also affected by the network of habitat patches that characterises small-holder farming communities [23], where depleted patches can quickly be recolonised from surrounding areas. Rodent control in such areas should follow a meta-population approach where rodent control is coordinated among several patches to limit individual patch recolonization [62]. We found no published and available studies that evaluated coordinated control strategies, highlighting the lack of coordinated efforts in rodent control. Our results here concur with others that have indicated that current control methods by small-holder farmers for rodent pests seem to be inadequate [23, 63] and where there has been success in Africa (e.g. [27] and Asia [64] there has been a strong emphasis on community campaigns [65].

Results from the predation studies were inconclusive. The majority of predation studies focussed on birds of prey, which have elsewhere produced some positive effects on rodent pest control [66]. However, our review found little to no support that increased avian predation reduced rodent populations to acceptable levels. The only observed effect detected was higher peak densities and faster population growth rates in the absence of predators [67]. These results concur with a recent meta-analysis on the effect of predation on prey populations [29], which suggest that rodents adapt their foraging behaviour according to predation risk, which has indeed be shown in *M*. *natalensis* [68]. These results here are however in contrast to a recent world-wide meta-analysis, which found a detectable negative effect of avian predation on rodent pests [69]. This discrepancy highlights the paucity of studies that makes it difficult to calculate effect sizes needed to estimate the magnitude of intervention effects [37]. Lastly, some studies suggested the use of domestic cats as a rodent control method. While domestic cats have been cited as rodent control agents [70], their effectiveness in rodent pest control is debateable [71]. In contrast, domestic cat diet seems to be dominated by native sylvatic animals (mammalian, reptilian and avian) which could actually impede rodent pest control, and care should be taken in advocating cats as effective pest control agents [72]. Furthermore, cats preying on rodents can be infected with *Toxoplama gondii* and in turn pose a significant health risk to humans [20].

### Trends in rodent pest research and species involved

Our analysis has highlighted that even though there has been a recent increase in the number of publications on rodent pest research, on an annual basis the number of papers are still few. Furthermore, research was highly restricted to only just a few African countries or sites within countries. There may be several reasons for low interest in rodent pest research related to agriculture. First, research is normally undertaken by researchers holding academic posts where pest control research may not be considered as a field producing high impact publications, a metric used to gauge academic performance [73]. Secondly, our review highlighted that well-funded large cross country collaborative projects (e.g. STAPLERAT; [74]; EcoRat; [26] are scientifically productive and may be the most appropriate avenues to establish long term projects which are required to produce robust estimates of abundance of rodents, and their damage and losses, with adequate spatial and temporal replication.

We observed a discrepancy in research themes between Tanzania and the rest of Africa. Tanzania generated the most research output due to the Pest Management Centre at Sokoine University of Agriculture, which also host the IRPM & BTD (African Centre of Excellence for Innovative Rodent Pest Management and Biosensor Technology Development). Tanzania therefore focussed more on applied research, compared with the rest of Africa that focussed on basic research. Researchers in Tanzania were ideally positioned to study the long history of rodent outbreaks in Africa, with specific focus into rodent outbreaks and its impact on crops [75, 76]. Over the 40 years Tanzanian researchers have invested heavily in understanding the ecology of its most important rodent pest, *Mastomys natalensis* [75], and with such understanding were able to investigate various interventions and modelling approaches to predict and manage crop losses [61, 77–80]. Of greatest concern is the fact that research regarding long-term rodent management is completely lacking from the rest of Africa.

One issue that is rarely considered is the balance of managing rodent pest species in an agricultural context whilst concurrently conserving those rodent species that occur in these landscapes and are not significant agricultural pests. In Africa, only about 5% of rodent species (up to 20 pest species out of 381 rodent species) are significant agricultural pests [59, 81]. The non-pest species often provide an important ecosystem service (see [82] for a review) and therefore need to be protected.

## Conclusions and recommendations

The overall objective of pest research is to reduce crop losses by achieving long-term sustainable population suppression of the key pest species [83, 84]. As such, the focus area of many studies has been population ecology, and our analysis highlights the significant challenges ecologists face in understanding population regulation, or at least how to manipulate it with management. Nonetheless, we highlight several recommendations that we feel will improve future studies and the evaluation of interventions:

1. We encourage researchers to adopt a ‘meta-analytic’ thinking framework when establishing intervention/management related research [37]. We suggest that researchers report effect statistics (and confidence intervals; see Nakagawa and Cuthill 2007 for details) which will quantify the size of experimental effects (e.g. mean difference). Standardised effect statistics are also dimensionless, which means independent studies (or treatment actions) can be compared. Furthermore, adopting this approach will facilitate future meta-analysis. For example, we found that studies rarely reported vital information such as sample sizes and standard deviations needed for meta-analysis.
2. We encourage researchers and funding organisations to establish and fund long-term studies [85]. Our analysis shows that only once a firm foundation has been established on the population ecology of the dominant species, other important aspects like management and community ecology can be successfully developed [75, 85].
3. We suggest that researchers adapt a unified or standardised approach to quantify rodent crop damage at the different cropping stages (e.g. [53, 86]. However, what is equally important is to relate crop damages to crop losses (yield losses). As such, more research is needed to accurately translate crop damages to crop (yield) losses, especially for different crops and cropping stages. Finally, it is also important to detail how crop damage was identified and if crop damage can be separated between different pest species.
4. We highlighted the paucity of research relating rodent density or abundance to crop damage [35]. This relationship is a key factor in determining if management intervention is needed, and yet little information pertaining to this relationship exists.
5. Rodent populations are often reported as capture success or minimum number of individuals alive, which can essentially be described as indices [87]. Ecologists need robust data to make informed management decisions [88], and we suggest that population data be analysed and reported in robust analytical frameworks (e.g. mark-recapture studies (88)).
6. There needs to be more empirical treatment-control studies with replicates that investigate management actions on rodent pest populations and associated crop losses. Ideally, these should be linked with household surveys conducted before and after management was implemented.
7. Finally, we suggest that ecologically-based rodent management will benefit from linking rodent population ecology with food webs [85, 89]. For example, the effectiveness of predation in reducing rodent populations is often investigated in isolation (or in a single species). However, by measuring these processes using food webs or ecological networks it is possible to establish a quantifiable yardstick to judge the structure and function of an ecosystem. This can guide future research as to how these networks may provide an ecosystem service like predation.

Our analysis shows that there are some well-recognised impacts of rodent pests on agricultural production, and that there are generally consistent estimates in the amount of crop damage occurring due to rodents, albeit based on a limited number of studies from a limited number of African countries. However, despite the high perceived losses gathered from interviews, backed up by a few systematic trials, replicated studies investigating sustainable rodent control solutions are few and far between. These gaps in research continue to have a significant impact on the development of ecologically-based rodent management strategies. Socio-economic benefits of sustainable rodent management are urgently required to improve food security for small-holder farmers in Africa and elsewhere where hunger, poverty and rodent pests are entwined.

## Supporting information

S1 Table PRISMA checklist. (PDF)

S2 Table List of rodent genera detected in rodent pest research in African agricultural systems from 1960–2015. (PDF)

S3 Table List of different crops and cropping system as impacted by rodent pests in African agriculture (1960–2015). (PDF)

S1 List Complete list of all publications used in the review–Publications in bold did not have full texts available at time of review. (DOCX)

S2 List Web of Science ^TM^ search history–. (TXT)

S1 Web of ScienceTM saved search (UA)
