# Correction: A systematic review of rodent pest research in Afro-Malagasy small-holder farming systems: Are we asking the right questions?

**DOI:** 10.1371/journal.pone.0176621

**Published:** 2017-04-20

**Authors:** Lourens H. Swanepoel, Corrie M. Swanepoel, Peter R. Brown, Seth J. Eiseb, Steven M. Goodman, Mark Keith, Frikkie Kirsten, Herwig Leirs, Themb’alilahlwa A. M. Mahlaba, Rhodes H. Makundi, Phanuel Malebane, Emil F. von Maltitz, Apia W. Massawe, Ara Monadjem, Loth S. Mulungu, Grant R. Singleton, Peter J. Taylor, Voahangy Soarimalala, Steven R. Belmain

There is an error in the affiliation of the sixteenth author. Grant R. Singleton is not affiliated with **13**. Grant R. Singleton is affiliated with **12**, Crop & Environmental Sciences Division, International Rice Research Institute, Metro Manila, Philippines, and **14** Natural Resources Institute, University of Greenwich, Chatham Maritime, Kent ME4 4TB, United Kingdom.

## References

[1] Swanepoel LH, Swanepoel CM, Brown PR, Eiseb SJ, Goodman SM, Keith M, et al (2017) A systematic review of rodent pest research in Afro-Malagasy small-holder farming systems: Are we asking the right questions? PLoS ONE 12(3): e0174554 doi:10.1371/journal.pone.0174554 28358899 10.1371/journal.pone.0174554 PMC5373544

