# Supplementary material for: A systematic review of rodent pest research in Afro-Malagasy small-holder farming systems: Are we asking the right questions?
Source: PLoS One. 2017 Mar 30;12(3):e0174554. doi: 10.1371/journal.pone.0174554 (PMC5373544; doi:10.1371/journal.pone.0174554)
Supplement: S2 Table — (PDF) [file pone.0174554.s002.pdf]

S2 Table List of rodent genera detected in rodent pest research in African agricultural systems from 1960 – 2015

Genus	Number of studies
Mastomys	70
Arvicanthis	42
Rattus	38
Mus	27
Lemniscomys	14
Gerbilliscus	18
Cricetomys	11
Meriones	10
Thryonomys	9
Xerus	9
Acomys	7
Dasymus	7
Rhabdomys	7
Hystrix	6
Lophuromys	6
Graphiurus	5
Praomys	5
Saccostomus	5
Steatomys	5
Taterillus	5
Aethomys	4
Thallomys	4
Atherurus	3
Grammomys	3
Paraxerus	3
Pelomys	3
Stenocephalemys	3
Thamnomys	3
Uranomys	3
Crocidura	2
Funisciurus	2
Heliosciurus	2
Malacomys	2
Oenomys	2
Anomalurus	1
Cryptomys	1
Dendromus	1
Desmodilliscus	1
Heterocephalus	1

Hlinsciurns	1
Hybomys	1
Hylomyscus	1
Jaculus	1
Nesokia	1
Otomys	1
Pachyuromys	1
Protoxerus	1
Psammonys	1
Sciuridae	1
Spalax	1
Tachyoryctes	1
