# Supplementary material for: A systematic review of rodent pest research in Afro-Malagasy small-holder farming systems: Are we asking the right questions?
Source: PLoS One. 2017 Mar 30;12(3):e0174554. doi: 10.1371/journal.pone.0174554 (PMC5373544; doi:10.1371/journal.pone.0174554)
Supplement: S3 Table — (PDF) [file pone.0174554.s003.pdf]

S3 Table List of different crops and cropping system as impacted by rodent pests in African agriculture (1960-2015)

Crop list	Nr of studies	Prop	Crop Category list	Nr of studies	Prop
Maize	55	0.22	Grain	128	0.56
Rice	21	0.08	Root vegetable	22	0.10
Wheat	17	0.07	Fruit tree	17	0.07
Crops	16	0.06	Vegetables	16	0.07
Cassava	10	0.04	Sugarcane	9	0.04
Sorghum	10	0.04	Groundnut	8	0.04
Grain	9	0.04	Legume	8	0.04
Sugarcane	9	0.04	Cacao	6	0.03
Groundnut	8	0.03	Coconut	4	0.02
Millet	8	0.03	Cotton	3	0.01
Beans	7	0.03	Palm	3	0.01
Barley	6	0.02	Fodder	2	0.01
Cacao	6	0.02	Coffee	1	0.00
Coconut	4	0.02	Plantation	1	0.00
Fallow	4	0.02			
Legumes	4	0.02			
Vegetables	4	0.02			
Yams	4	0.02			
Banana	3	0.01			
Cotton	3	0.01			
Fruit	3	0.01			
Palm	3	0.01			
Potato	3	0.01			
Root crops	3	0.01			
Cowpea	2	0.01			
Lucerne	2	0.01			
Olive	2	0.01			
Orchard	2	0.01			
Sweet potato	2	0.01			
Tomato	2	0.01			
Almond	1	0.00			
Banana	1	0.00			
Cabbage	1	0.00			
Carob	1	0.00			
Citrus	1	0.00			
Coffee	1	0.00			
Dates	1	0.00			
Enset	1	0.00			
Grass	1	0.00			
Lentils	1	0.00			
Onions	1	0.00			

Pea	1	0.00
Plantain	1	0.00
Plantation	1	0.00
Pumpkin	1	0.00
Sesame	1	0.00
