# Supplementary material for: A systematic review of rodent pest research in Afro-Malagasy small-holder farming systems: Are we asking the right questions?
Source: PLoS One. 2017 Mar 30;12(3):e0174554. doi: 10.1371/journal.pone.0174554 (PMC5373544; doi:10.1371/journal.pone.0174554)
Supplement: S1 List — (DOCX) [file pone.0174554.s004.docx]

S1 List. Complete list of all publications used in the review – Publications in bold did not have full texts available at time of review.

**1. Ali MK, Farghal AI (1995) Damage caused by rodents to sugar cane varieties and juice quality in Sohag Governorate. Assiut Journal of Agricultural Sciences 26: 231-237.**

**2. Asran AA (1993) Protection of some fruits against the climbing rat, *Rattus rattus* damage by using certain means in Beheira Governorate. Egyptian Journal of Agricultural Research 71: 915-920.**

**3. Asran AA, El-Deeb HI, El-Halafaway MA (1991) Rat damage to certain crops and population density of Arvicanthis niloticus in Fayoum Governorate. Egyptian Journal of Agricultural Research 69: 273-280.**

**4. Bellier L (1973) Rodent pests of West Africa. SPAN Shell Publ Health Agr News.**

**5. Delattre P, Duplantier JM, Fichet-Calvet E, Giraudoux P (1998) Environmental modifications and rodent outbreaks: impact on agriculture and public health. Cahiers d'Etudes et de Recherches Francophones Agricultures (France).**

**6. Dickman CR (2003) Rodent-ecosystem relationships: a review. In: Singleton G, Hinds L, Leirs H, Zhang Z, editors. Ecologically-based Management of Rodent Pests: ACIAR Monograph No. 59 Australian Centre For International Agricultural Research: Canberra.**

**7. El-Deeb HI, Asran AA, El-Halafawy MA (1990) Pre-harvest damage and active burrows of rats in wheat fields. Agricultural Research Review 68: 229-233.**

**8. El-Deeb HI, Zidan ZH, El-Hawashy N, Mourad AA (1999) Survey studies on rodent fauna of the new reclaimed area and their role on crop damage in Egypt. Annals of Agricultural Science (Cairo) 44: 775-790.**

**9. Fayenuwo JO, Akande M. The Economic Importance and Control of Cane-Rat (Thryonomys swinderianus Temmick); 2002. pp. 86-90.**

**10. Fiedler LA, Fall MW (1994) Rodent control in practice: tropical field crops.**

**11. Funmilayo O (1973) A general survey of the incidences and control methods of vertebrate pests of crop plants in Western State of Nigeria. Research Bulletin, University of Ife Institute of Agricultural Research and Training: 40pp.-40pp.**

**12. Hubert B, Adam F (1985) Outbreaks of Mastomys erythroleucus and Taterillus gracilis in the Sahelo-Sudanian zone in Senegal. Acta Zoologica Fennica 173: 113-117.**

**13. Key G (1990) Preharvest crop losses to the African striped ground-squirrel, Xerus erythropus, in Kenya. Tropical Pest Management 36: 223-229.**

**14. Kuehnert GF (1986) Estimation of rodent damage in tomato crop in Upper and Middle Egypt. Acta Horticulturae: 139-143.**

**15. Mkondya CB (1977) Preliminary proposals and hints for approaches to outbreak evaluation and control strategies against heavy rodent infestations in the Shinyanga outbreak foci in Tanzania. Dar es Salaam, Tanzania, Ministry of Agriculture, Crop Development Division.**

**16. Mmetwaly AM, Montasser SA, Al-Gendy AAR (2009) Survey of rodent species and damage assessment caused by Meriones shawi isis (Thomas) in some field crops at Bustan area. Journal of Applied Sciences Research: 40-45.**

**17. Mulungu LS (2003) Assessment of maize (Zea mays L.) damage and yield loss due to rodents in the field. Morogoro, Tanzania: Sokoine University of Agriculture. 178 p.**

**18. Myllymaki A (1989) Denmark-Tanzania Rodent Control Project - Final Report (unpublished), Rodent Control Centre, Morogoro, Tanzania.**

**19. Myllymäki A (1979) Importance of small mammals as pests in agriculture and stored products. Ecology of small mammals: Springer. pp. 239-279.**

**20. Ohiagu CC (1987) Storage of food grains in the savannah zones of Northern Nigeria. Food Grain Production in Semi Arid Africa, SAFGRAD, Ouagadougou, Burkina Faso: 361-368.**

**21. Olakojo SA (2001) Comparative assessment of vertebrate pest damage on some maize varieties in South-western Nigeria. Tropical Agricultural Research and Extension 4: 112-114.**

**22. Pomela ML, Sheriff R. The significance of pests in the pre and post production system of sorghum in Lesotho food security; 1988 1988. pp. 31-38.**

**23. Rady GH, Asran AA, Ramzy B (2001) Survey and damage appraisal of rodents in certain field crops in Kalubia governorate. Annals of Agricultural Science, Moshtohor 39: 1779-1786.**

**24. Raymundo SA (1984) Traditional methods of rice pest control in Sierra Leone, West Africa. WARDA Technical Newsletter 5: 23-24.**

**25. Sanchez FF. Rodents affecting food supplies in developing countries-Problems and needs and Public Health; 1975.**

**26. Segeren P, Van Den Oever R, Slobbe W (1995) Seasonal abundance, damage, cultural control methods and varietal resistance of the four main pest and disease problems in irrigated maize in southern Mozambique. Insect Science and its Application 16: 263-277.**

**27. Smith RH (1994) Rodents and birds as invaders of stored-grain ecosystems. Stored-Grain Ecosystems; Jayas, DS, White, NDG, Muir, WE, Eds: 289-323.**

**28. Tanaka A, Saito K, Azoma K, Kobayashi K (2013) Factors affecting variation in farm yields of irrigated lowland rice in southern-central Benin. European Journal of Agronomy 44: 46-53.**

**29. Taylor KD (1961) An investigation of damage to West African cocoa by vertebrate pests. Unpublished internal report, Ministry of Agriculture, Tolworth, England.**

**30. Taylor KD, Green MG (1972) An ecological study of the rodent pests of cereals in the Kenya Highlands. Unpublished report Pest Infestation Control Laboratory, Ministry of Agriculture, Fisheries and Food, Tolworth, Surrey, UK.**

**31. Tristiani H, Murakami O, Kuno E (2000) Rice plant damage distribution and home range distribution of the ricefield rat Rattus argentiventer (Rodentia: Muridae). Belgian Journal of Zoology 130: 83-91.**

**32. Van Gulck T, Stoks R, Verhagen R, Sabuni CA, Mwanjabe P, et al. (1998) Short-term effects of avian predation variation on population size and local survival of the multimammate rat, Mastomys natalensis (Rodentia, Muridae). Mammalia 62: 329-340.**

**33. Walker PT. A survey of losses of cereals to pests in Kenya and Tanzania; 1967.**

34. Stenseth NC, Leirs H, Mercelis S, Mwanjabe P (2001) Comparing strategies for controlling an African pest rodent: an empirically based theoretical study. Journal of Applied Ecology 38: 1020-1031.

35. Massawe AW, Rwamugira W, Leirs H, Makundi RH, Mulungu LS (2007) Do farming practices influence population dynamics of rodents? A case study of the multimammate field rats, Mastomys natalensis, in Tanzania. African Journal of Ecology 45: 293-301.

36. Rasamoelina G, Rasamoel M, Rakotovao J-M, Rafanomezana S (1997) Resultats d'une évaluation des dégâts de rats sur le riz irrigué á Madagascar pendant la saison 1996-1997 (Results of an evaluation of rat damage in irrigated rice in Madagascar). Ronguers et Lutte Antimurine a Madagascar.

37. Hubert B, Adam F (1983) The regulation of the population dynamics of two sahelian rodents in Senegal: an hypothesis. Annales-Musee Royal de l'Afrique Centrale-Sciences Zoologiques (Belgium) 237: 193-201.

38. Sichilima AM, Zulu MS, Leirs H (2003) The effects of Tephrosia vogelii and land preparation methods on mole rat activity in cassava fields. ACIAR MONOGRAPH SERIES 96: 254-255.

39. Rabiu S, Rose RK (2004) Crop damage and yield loss caused by two species of rodents in irrigated fields in northern Nigeria. International Journal of Pest Management 50: 323-326.

40. Makundi RH, Massawe AW (2011) Ecologically based rodent management in Africa: potential and challenges. Wildlife Research 38: 588-595.

41. Bernard J (1977) Damage caused by the rodents Gerbillidae to agriculture in North Africa and countries of the Middle East. EPPO Bulletin 7: 283-296.

42. Ngowo V, Lodal J, Mulungu LS, Makundi RH, Massawe AW, et al. (2003) Evaluation of thiram and cinnamamide as potential repellents against maize-seed depredation by the multimammate rat, Mastomys natalensis, in Tanzania. ACIAR MONOGRAPH SERIES 96: 260-261.

43. Granjon L, Traoré M (2007) Prey selection by barn owls in relation to small-mammal community and population structure in a Sahelian agro-ecosystem. Journal of Tropical Ecology 23: 199-208.

44. Mohr K, Vibe‐Petersen S, Lau Jeppesen L, Bildsøe M, Leirs H (2003) Foraging of multimammate mice, Mastomys natalensis, under different predation pressure: cover, patch‐dependent decisions and density‐dependent GUDs. Oikos 100: 459-468.

45. Ojwang DO, Oguge NO (2003) Testing a biological control program for rodent management in a maize cropping system in Kenya. In: Singleton G, Hinds L, Krebs C, Spratt DM, editors. ACIAR Monograph Series 96: Australian Centre for International Agricultural Research (ACIAR). pp. 251-253.

46. Naughton‐Treves L (1998) Predicting patterns of crop damage by wildlife around Kibale National Park, Uganda. Conservation biology 12: 156-168.

47. Taylor KD (1968) An Outbreak of Rats in Agricultural Areas of Kenya in 1962. East African Agricultural and Forestry Journal 34: 66-77.

48. Barnett AA, Read N, Scurlock J, Low C, Norris H, et al. (2000) Ecology of rodent communities in agricultural habitats in eastern Sierra Leone: Cocoa groves as forest refugia. Tropical Ecology 41: 127-142.

49. Osborn FV, Hill CM (2005) Techniques to reduce crop loss: human and technical dimensions in Africa. Conservation Biology Series (Cambridge) 9: 72.

50. Makundi RH, Oguge NO, Mwanjabe PS (1999) Rodent pest management in East Africa - an ecological approach. In: Singleton GR, Hinds LA, Leirs H, Zhang Z, editors. Ecologically-based management of rodent pests. pp. 460-476.

51. Meheretu Y, Sluydts V, Welegerima K, Bauer H, Teferi M, et al. (2014) Rodent abundance, stone bund density and its effects on crop damage in the Tigray highlands, Ethiopia. Crop Protection 55: 61-67.

52. Makundi RH, Massawe AW, Laswai HS, Makundi RH (2006) Storage and protection of Durable Food crops and their products in Tanzania. In: Makundi R, editor. Management of selected crop pests in Tanzania Dar es Salaam: Tanzania Publishing House. pp. 185-204.

53. Zehrer W (1997) La grande invasion murine de 1965 a Madagascar (The big rodent invasion in Madagascar in 1965). Ronguers et Lutte Antimurine a Madagascar.

54. Datiko D, Bekele A, Belay G (2008) Feeding ecology of pest rodents from Arbaminch forest and farmlands, Ethiopia. SINET: Ethiopian Journal of Science 30: 127-134.

55. Massawe AW, Rwamugira W, Leirs H, Makundi RH, Mulungu LS, et al. (2008) Soil type limits population abundance of rodents in crop fields: case study of the multimammate rat Mastomys natalensis Smith, 1834 in Tanzania. Integrative zoology 3: 27-30.

56. Bekele A, Leirs H (1997) Population ecology of rodents of maize fields and grassland in central Ethiopia. Belgian Journal of Zoology (Belgium) 127: 39-48.

57. Oerke E-C (2006) Crop losses to pests. The Journal of Agricultural Science 144: 31-43.

58. Jackson WB (1977) Evaluation of Rodent Depredations to Crops and Stored Products1. EPPO Bulletin 7: 439-458.

59. Odhiambo RO, Makundi RH, Leirs H, Verhagen R (2005) Community structure and seasonal abundance of rodents of maize farms in southwestern Tanzania. Belgium Journal of Zoology 135: 113-118.

60. Green MG, Taylor KD (1975) Preliminary experiments in habitat alteration as a means of controlling field rodents in Kenya. Ecological Bulletins: 175-186.

61. Van Hooft P, Cosson J-F, Vibe‐Petersen S, Leirs H (2008) Dispersal in Mastomys natalensis mice: use of fine‐scale genetic analyses for pest management. Hereditas 145: 262-273.

62. Skonhoft A, Leirs H, Andreassen HP, Mulungu LSA, Stenseth NC (2006) The bioeconomics of controlling an African rodent pest species. Environment and Development Economics 11: 453-475.

63. Meheretu Y, Kiros W, Seppe D, Raes D, Makundi RH, et al. (2010) Farmers' perspectives of rodent damage and management from the highlands of Tigray, Northern Ethiopian. Crop Protection 29: 532-539.

64. Fayenuwo JO, Olakojo SA, Ak M, Amusa NA, Olujimi OA (2007) Comparative evaluation of vertebrate pest damage on some newly developed quality protein maize (QPM) varieties in south-western Nigeria. African Journal of Agricultural Research 2: 592-595.

65. Poulet A (1980) The 1975-76 rodent outbreak in a northern Senegal irrigated farmland. Biotrop Special Publication: 123-138.

66. Shenkut M, Mebrate A, Balakrishnan M (2006) Distribution and abundance of rodents in farmlands: a case study in Alleltu Woreda, Ethiopia. SINET: Ethiopian Journal of Science 29: 63-70.

67. Vibe-Petersen S, Leirs H, Bruyn LD (2006) Effects of predation and dispersal on Mastomys natalensis population dynamics in Tanzanian maize fields. Journal of Animal Ecology 75: 213-220.

68. Leirs H, Sluydts V, Makundi RH (2010) Rodent outbreaks in sub-Saharan Africa. In: Singleton G, Belmain S, Brown PR, Hardy B, editors. Rodent Outbreaks: Ecology and Impacts. pp. 269-280.

69. Eisen RJ, Enscore RE, Atiku LA, Zielinski-Gutierrez E, Mpanga JT, et al. (2013) Evidence that rodent control strategies ought to be improved to enhance food security and reduce the risk of rodent-borne illnesses within subsistence farming villages in the plague-endemic West Nile region, Uganda. International Journal of Pest Management 59: 259-270.

70. Makundi RH, Massawe AW (2003) Review of recent advances in studies of the ecology of Mastomys natalensis (Smith 1834)(Rodentia: Muridae) in Tanzania, eastern Africa. ACIAR MONOGRAPH SERIES 96: 242-245.

71. Barre MH (1978) Control campaign against a rat outbreak in Somalia with special reference to the Shebelli and Juba Valley agricultural areas. Unpubl Rep p: 1-9.

72. Funmilayo O (1982) Assessment of rodent damage in field rice and rodent control procedures. Integrated Pest Management in Rice in West Africa WARDA Regional Training Center, FenDall, Liberia.

73. de Lima CPF (1979) The assessment of losses due to insects and rodents in maize stored for subsistence in Kenya. Tropical Stored Products Information 38: 21-26.

74. Mulungu LS, Lagwen PP, Mdangi ME, Kilonzo BS, Belmain SR (2014) Impact of spatio-temporal simulations of rat damage on yield of rice (Oryza sativa L.) and implications for rodent pest management. International Journal of Pest Management 60: 269-274.

75. Fiedler LA (1988) Rodent problems in Africa. Rodent pest management: 35-65.

76. Leirs H, Makundi RH, Davis S (2005) The present issue of the Belgian Journal of Zoology contains the proceedings of the 9th International African Small Mammal Symposium (ASMS), held at the Sokoine University of Agriculture, Morogoro, Tanzania from 14-18 July 2003. Belg J Zool 135: 3.

77. Sidorowicz J (1974) Rodents feeding on cassava, Manihot esculenta Granz.[sic]. Mammalia 38: 344-346.

78. Sluydts V, Davis S, Mercelis S, Leirs H (2009) Comparison of multimammate mouse (Mastomys natalensis) demography in monoculture and mosaic agricultural habitat: Implications for pest management. Crop Protection 28: 647-654.

79. Funmilayo O (1980) Mammals and birds affecting food production and storage in Nigeria.

80. Belmain SR, Meyer AN, Timbrine R, Penicela L (2003) Managing rodent pests in households and food stores through intensive trapping. ACIAR MONOGRAPH SERIES 96: 440-445.

81. Fiedler LA ( 1985) The status of rodent control in five east African countries. FAO Report.

82. Key GE. Control of the African striped ground squirrel, Xerus erythropus, in Kenya; 1990. pp. 48.

83. Mdangi M, Mulungu LS, Massawe AW, Eiseb SJ, Tutjavi V, et al. (2013) Assessment of rodent damage to stored maize (Zea mays L.) on smallholder farms in Tanzania. International Journal of Pest Management 59: 55-62.

84. Rasamoel M, Rafanomezana S (1997) Protection des dentrees stockees contre les rats en mulieu rural et en mulieu urbain - Cas d'Antananarivo et des environs de Moramanga (Protection of stored produce against rats in rural and urban areas - Case of Antananarivo and the vicinity of Moramanga). Ronguers et Lutte Antimurine a Madagascar.

85. Belmain SR, Dlamini N, Eiseb SJ, Kirsten F, Mahlaba T, et al. (2008) The ECORAT project: developing ecologically-based rodent management for the southern African Region. International Pest Control 50: 136-138.

86. Ezealor AU, Giles Jr RH (1997) Vertebrate pests of a Sahelian wetland agro-ecosystem: perceptions and attitudes of the indigenes and potential management strategies. International Journal of Pest Management 43: 97-104.

87. Fiedler LA (1987) An assessement of the current rodent outbreak in Sudan. International Program Section Unpublished report.

88. Takele S, Bekele A, Belay G, Balakrishnan M (2008) Pest status of rodents in Wonji sugarcane plantation, Ethiopia. Int J Ecol Environ Sci 34: 157-163.

89. Akande M (1986) The economic importance and control of vertebrae pests of graminaceous crops with particular reference to rice (Oryza sativa) in Nigeria - A Review. Proceedings of the Twelfth Vertebrate Pest Conference.

90. Fieldler LA (1988) Rodent pest problems and management in eastern Africa. FAO Plant Protection Bulletin 36: 125-134.

91. Mulungu LS, Ngowo V, Mdangi M, Katakweba AS, Tesha P, et al. (2013) Population dynamics and breeding patterns of multimammate mouse, Mastomys natalensis (Smith 1834), in irrigated rice fields in Eastern Tanzania. Pest management science 69: 371-377.

92. Stenseth NC, Leirs H, Skonhoft A, Davis SA, Pech RP, et al. (2003) Mice, rats, and people: the bio-economics of agricultural rodent pests. Frontiers in Ecology and the Environment 1: 367-375.

93. Smith RH, Nott HMR (1988) Rodent damage to cocoa in Equatorial Guinea. FAO Plant Protection Bulletin 36: 119-124.

94. Naughton Treves L (1997) Farming the forest edge: vulnerable places and people around Kibale National Park, Uganda. Geographical Review 87: 27-46.

95. Datiko D, Bekele A (2013) Conservation Challenge: Human-herbivore Conflict in Chebera Churchura National Park, Ethiopia. Pakistan Journal of Biological Sciences 16: 1758-1764.

96. Granjon L, Cosson J-F, Quesseveur E, Sicard B (2005) Population dynamics of the multimammate rat Mastomys huberti in an annually flooded agricultural region of central Mali. Journal of Mammalogy 86: 997-1008.

97. Rafanomezana S (1997) Les rats dans les cultures maraicheres - Description des degats sur differentes speculations et nuisibilite a l'exemple du petit pois (Rats in vegetable crops - Symptom descriptions on several vegetable species and damage at the example of peas). Ronguers et Lutte Antimurine a Madagascar.

98. Asimalowo AA, Ayodele AI, Singh BN (1998) Rodent pests of upland and lowland rice at a derived savanna site in Nigeria. International Rice Research Notes 23: 29-29.

99. Odhiambo RO, Makundi RH, Leirs H, Verhagen R (2008) Demography, reproductive biology and diet of the bushveld gerbil Tatera leucogaster (Rodentia: Gerbillinae) in the Lake Rukwa valley, south‐western Tanzania. Integrative zoology 3: 31-37.

100. Monadjem A, Mahlaba TaA, Dlamini N, Eiseb SJ, Belmain SR, et al. (2011) Impact of crop cycle on movement patterns of pest rodent species between fields and houses in Africa. Wildlife Research 38: 603-609.

101. Makundi RH, Mbise TJ, Kilonzo BS (1991) Observations on the role of rodents in crop losses in Tanzania and control strategies. Beiträge zur tropischen Landwirtschaft und Vertirinärmedizin 29: 465-474.

102. Singleton GR, Leirs H, Hinds LA, Zhang Z (1999) Ecologically-based management of rodent pests–re-evaluating our approach to an old problem. In: Singleton GR, Leirs H, Hinds LA, Zhang Z, editors. Ecologically-based Management of Rodent Pests Australian Centre for International Agricultural Research (ACIAR), Canberra. pp. 17-29.

103. Kennis J, Sluydts V, Leirs H, van Hooft WF (2008) Polyandry and polygyny in an African rodent pest species, Mastomys natalensis. Mammalia 72: 150-160.

104. Taylor K (1976) An outline of the rodent pest problem in Tanzania. Unpublished document on a two week visit in 1976 Presented to the Ministry of Agriculture, Dar es Salaam, Tanzania.

105. Cudjoe AR (2009) Vertebrate Pests of Cassava in Africa Their Control. African Crop Science Journal 2: 497-503.

106. Belmain SR, Meyer AN, Penicela L, Xavier R. Population management of rodent pests through intensive trapping inside rural households in Mozambique; 2002.

107. Kasso M (2013) Pest Rodent Species Composition, Level of Damage and Mechanism of control in Eastern Ethiopia. International Journal of Innovation and Applied Studies 4: 502-511.

108. Makundi RH, Massawe AW, Mulungu LS (2007) Reproduction and population dynamics of Mastomys natalensis Smith, 1834 in an agricultural landscape in the Western Usambara Mountains, Tanzania. Integrative Zoology 2: 233-238.

109. Spragins CW (2002) Advances in IPM rodent control in agriculture. Sustain Dev Int, Earth Summit: 135-140.

110. Arlet ME, Molleman F (2007) Rodents damage crops more than wildlife in subsistence agriculture on the northern periphery of Dja Reserve, Cameroon. International Journal of Pest Management 53: 237-243.

111. Ogada DL, Kibuthu PM (2008) Conserving Mackinder's eagle owls in farmlands of Kenya: assessing the influence of pesticide use, tourism and local knowledge of owl habits in protecting a culturally loathed species. Environmental Conservation 35: 252-260.

112. Greaves JH (1989) Rodent pests and their control in the Near East. Paper 95.

113. Belmain S (2010) Developing pesticide-free rodent control for southern Africa. Pesticides News: 9-11.

114. Brown RZ (1970) Rodent control problems in developing countries. Proceedings of the 4th Vertebrate Pest Conference.

115. Mulungu LS, Makundi RH, Leirs H (2003) Robustness of techniques for estimating rat damage and yield loss in maize fields. ACIAR MONOGRAPH SERIES 96: 224-228.

116. Mwanjabe PS, Leirs H (1997) An early warning system for IPM-based rodent control in smallholder farming systems in Tanzania. Belgian Journal of Zoology 127: 49-58.

117. Duplantier J-M, Rakotondravony D (1999) The Rodent Problem in Madagascar: Agricultural Pest and Threat to Human Health. in: Ecologically-based rodent management Eds : Grant Singleton, Lyn Hinds, Herwig Leirs and Zhibin Zhang ACIAR editions.

118. Agona A, Nabawanuka J, Muyinza H (2001) An overview of maize in Uganda. Post-harvest Programme, NARO Uganda.

119. Massawe AW, Rwamugira W, Leirs H, Makundi RH, Mulungu LS (2005) Influence of land preparation methods and vegetation cover on population abundance of Mastomys natalensis in Morogoro, Tanzania. Belgian Journal of Zoology 135: 187-193.

120. Tyler PS, Boxall RA (1984) Post harvest loss reduction programmes: A decade of activities-what consequences? Tropical Stored Products Information 50: 4-13.

121. Diagne A, Alia D, Amovin-Assagba E, Wopereis MCS, Saito K, et al. (2013) Farmer perceptions of the biophysical constraints to rice production in sub-Saharan Africa, and potential impact of research. Realizing Africa's Rice Promise: 46-68.

122. Fiedler LA (1990) Rodent pests in Guinea-Bissau. Denver Wildlife Research Center. Unplished report prepared for the United States Agence for International Development. International program research section.

123. von Maltitz EF, Kirsten F, Malebana PS, Belmain SR, Sandmann E, et al. (2003) Developing a rodent management strategy for South Africa's Limpopo province. In: Singleton G, Hinds L, Krebs C, Sprat DM, editors. ACIAR Monograph Series Nr 96. pp. 418-421.

124. Kirsten F, von Maltitz E (2005) Technology transfer and promotion of ecologically-based and sustainable rodent control strategies in South Africa R 8190 (ZA 0506). Final technical report. Agricultural Research Council, Pretoria.

125. Mulungu LS, Makundi RH, Massawe AW, Leirs H (2007) Relationship between sampling intensity and precision for estimating damage to maize caused by rodents. Integrative Zoology 2: 131-135.

126. Mulungu LS, Makundi RH, Leirs H, Massawe AW, Vibe-Petersen S, et al. (2003) The rodent density-damage function in maize fields at an early growth stage. In: Singleton GR, Hinds LA, Krebs CJ, Spratt DM, editors. ACIAR Monograph Series No 96, 564p. pp. 301-303.

127. Smythe WR. Rodent control in East Africa. In: Salmon TP, editor; 1986. Printed at Univ. of California, Davis, Calif.

128. Leirs H, Verhagen R, Verheyen W, Mwanjabe P, Mbise T (1996) Forecasting rodent outbreaks in Africa: an ecological basis for Mastomys control in Tanzania. Journal of Applied Ecology: 937-943.

129. Massawe AW, Leirs H, Rwamugira WP, Makundi RH (2003) Effect of land preparation methods on spatial distribution of rodents in crop fields. ACIAR MONOGRAPH SERIES 96: 229-232.

130. Belmain SR (2007) Rats: an ecologically-based approach for managing a global problem. LEISA Magazine 23: 18-20.

131. Makundi RH, Bekele A, Leirs H, Massawe AW, Rwamugira W, et al. (2005) Farmer’s perceptions of rodents as crop pests: knowledge, attitudes and practices in rodent pest management in Tanzania and Ethiopia. Belgian Journal of Zoology 135: 153-157.

132. Leirs H (2003) Management of rodents in crops: the Pied Piper and his orchestra. ACIAR Monograph Series 96: 183-190.

133. Bekele A, Leirs H, Verhagen R (2003) Composition of rodents and damage estimates on maize farms at Ziway, Ethiopia. ACIAR MONOGRAPH SERIES 96: 262-263.

134. Takele S, Bekele A, Belay G, Balakrishnan M (2011) A comparison of rodent and insectivore communities between sugarcane plantation and natural habitat in Ethiopia. Tropical Ecology 52: 61-68.

135. Leirs H (1994) Population ecology of Mastomys natalensis (Smith, 1834): implications for rodent control in Africa. A report for the Tanzania-Belguim joint rodent research project (1986-1989)

136. Schill PF, Afreh-Nuamah K, Gold CS, Green KR (2000) Farmers' perceptions of constraints to plantain production in Ghana. The International Journal of Sustainable Development & World Ecology 7: 12-24.

137. Sluydts V, Crespin L, Davis S, Lima M, Leirs H (2007) Survival and maturation rates of the African rodent, Mastomys natalensis: density‐dependence and rainfall. Integrative Zoology 2: 220-232.

138. Singleton GR, Brown PR, Jacob J, Aplin KP (2007) Unwanted and unintended effects of culling: A case for ecologically‐based rodent management. Integrative zoology 2: 247-259.

139. Walker PT (1983) Crop losses: The need to quantify the effects of pests, diseases and weeds on agricultural production. Agriculture, Ecosystems & Environment 9: 119-158.

140. Drazo NA, Kennis J, Leirs H, Migimiru DA (2008) Farmer survey in the hinterland of Kisangani (Democratic Republic of Congo) on rodent crop damage and rodent control techniques used. Mammalia 72: 192-197.

141. Taylor PJ, Downs S, Monadjem A, Eiseb SJ, Mulungu LS, et al. (2012) Experimental treatment-control studies of ecologically based rodent management in Africa: balancing conservation and pest management. Wildlife Research 39: 51-61.

142. Ali AM (1978) The changing rodent pest fauna in Egypt. Proceedings of the Vertebrate Pest Conference 8: 28-31.

143. Gratz NG, Arata AA (1975) Problems associated with the control of rodents in tropical Africa. Bulletin of the World Health Organization 52: 697.

144. Davis SA, Leirs H, Pech RP, Zhang Z, Stenseth NC (2004) On the economic benefit of predicting rodent outbreaks in agricultural systems. Crop Protection 23: 305-314.

145. Funmilayo O, Akande M (1977) Vertebrate pests of rice in southwestern Nigeria. Pans 23: 38-48.

146. Mwanjabe PS, Sirima FB, Lusingu J (2002) Crop losses due to outbreaks of Mastomys natalensis (Smith, 1834) Muridae, Rodentia, in the Lindi Region of Tanzania. International Biodeterioration & Biodegradation 49: 133-137.

147. Adesina AA, Johnson DE, Heinrichs EA (1994) Rice pests in the Ivory Coast, West Africa: farmers’ perceptions and management strategies. International Journal of Pest Management 40: 293-299.

148. Everard COR (1966) Report on some aspects of rodent damage to maize in the western Region of Nigeria. Unpublished report to the Ministry of Agriculture and Natural Resources, Western Nigeria.

149. Delany MJ, Kansiimeruhanga WDK (1970) Observations on the ecology of rodents from a small arable plot near Kampala, Uganda. Review of Zoology and Botany in Africa 81: 417-425.

150. Mulungu LS, Themb'alilahlwa AM, Massawe AW, Kennis J, Crauwels D, et al. (2011) Dietary differences of the multimammate mouse, Mastomys natalensis (Smith, 1834), across different habitats and seasons in Tanzania and Swaziland. Wildlife Research 38: 640-646.

151. Leirs H, Verhagen R, Verheyen W. The use of rainfall patterns in predicting population densities of multimammate rats, Mastomys natalensis; 1990. pp. 53.

152. Singleton GR, Belmain SR, Brown PR (2010) Rodent outbreaks: an age-old issue with a modern appraisal. In: Singleton G, Belmain S, Brown P, Hardy B, editors. Rodent outbreaks: Ecology and impacts: IRRI. pp. 1-8.

153. Elias DJ (1988) Overview of rodent problems in developing countries. FAO Plant Protection Bulletin (FAO) 36: 107-110.

154. Meerburg BG, Singleton GR, Leirs H (2009) The Year of the Rat ends—time to fight hunger! Pest Management Science 65: 351-352.

155. Funmilayo O (1976) Vertebrate pest damage to maize ears and control recommendations. Nigerian Journal of Plant Protection 2: 15-18.

156. Dlamini N, Eiseb S, Kirsten F, Mahlaba T, Makundi R, et al. (2008) The ECORAT project: developing ecologically-based rodent management for the southern African region. International Pest Control 50: 136-138.

157. Tobin ME, Fall MW (2004) Pest control: rodents. USDA National Wildlife Research Center-Staff Publications: 67.

158. Tefera T (2012) Post-harvest losses in African maize in the face of increasing food shortage. Food security 4: 267-277.

159. Desoky A (2015) Pre-harvest Damage Caused by the Nile grass rat, Arvicanthis niloticus in Wheat Fields at Sohag region, Egypt. Academic Research Journal of Agricultural Science and Research and Exploration 3: 188-191.

160. Mulungu LS, Belmain SR, Dlamini N, Eiseb S, Kirsten F, et al. (2011) The Ecorat project: development of ecologically-based rodent management for the southern African region. Julius-Kuhn-Archiv: 165-166.

161. Chitere PO, Omolo BA (1993) Farmers’ indigenous knowledge of crop pests and their damage in western Kenya. International Journal of Pest Management 39: 126-132.

162. Odhiambo CO, Oguge NO (2003) Patterns in rodent pest distribution in a maize cropping system in the Kenyan Rift Valley. ACIAR MONOGRAPH SERIES 96: 217-219.

163. Yonas M, Welegerima K, Deckers S, Raes D, Makundi RH, et al. (2010) Farmers' perspectives of rodent damage and management from the highlands of Tigray, Northern Ethiopian. Crop Protection 29: 532-539.

164. Hill CM (1997) Crop-raiding by wild vertebrates: The farmer's perspective in an agricultural community in western Uganda. International Journal of Pest Management 43: 77-84.
